# Supplementary material for: Experimental realization of entangled coherent states in two-dimensional harmonic oscillators of a trapped ion
Source: Sci Rep. 2024 Mar 21;14:6847. doi: 10.1038/s41598-024-57391-6 (PMC11349922; doi:10.1038/s41598-024-57391-6)
Supplement: Supplementary file 1 — Supplementary Information. [file 41598_2024_57391_MOESM1_ESM.pdf]

Supplementary Material

Experimental Realization of Entangled Coherent States in Two-dimensional Harmonic Oscillators of a Trapped Ion

Honggi Jeon,[1,2] Jiyong Kang,[1,2] Jaeun Kim,[1,2] Wonhyeong Choi,[1,2,3] Kyunghye Kim,[1,2] and Taehyun Kim[2,1,3,4,5,*]

[1]*Department of Computer Science and Engineering, Seoul National University, Seoul 08826, Republic of Korea*

[2]*Automation and System Research Institute, Seoul National University, Seoul 08826, Republic of Korea*

[3]*Inter-university Semiconductor Research Center, Seoul National University, Seoul 08826, Republic of Korea*

[4]*Institute of Computer Technology, Seoul National University, Seoul 08826, Republic of Korea*

[5]*Institute of Applied Physics, Seoul National University, Seoul 08826, Republic of Korea*

I. PHONON DISTRIBUTION EXTRACTION PROCEDURE

In the experiments presented in this work, we chose to observe the variation in the phonon population distribution of the Y mode because it is less affected by off-resonant Rabi oscillation of the other motional mode. The angles between the momentum transfer vector the the laser beams and the X and Y principal axes are 66 degrees and 24 degrees, respectively. This results in uneven Lamb-Dicke factors, $\eta_X = 0.05$ and $\eta_Y = 0.11$. Therefore, when we drive the Y blue sideband Rabi oscillation, the amplitude of the X mode Rabi oscillation is $(\eta_X \Omega_0 \sqrt{n+1})^2/((\eta_X \Omega_0 \sqrt{n+1})^2 + \delta^2)$ where Ω_0 is the carrier Rabi frequency, n is the number of phonons and δ is the spacing between the X and Y modes. The amplitude is 0.005 when the Y mode is driven with $\Omega_0 = 2\pi * 40$ kHz and $n = 0$. When the X mode is driven with the same Rabi frequency, the amplitude of the off-resonant Rabi oscillation of the Y mode is 0.02 for $n = 0$ and reaches 0.11 when $n = 4$, the largest mean phonon number in our experiments. Therefore, we probed the Y mode population as it is expected to be more straight forward to analyze.

We used a simple exponential decay model of the following form to analyze the blue-sideband Rabi oscillation results shown in Fig. 3 of the main text [1–3].

$$P_{\uparrow}(t_{BSB}) = \sum_{n=0}^{N} \frac{p_{Y,n}}{2} \left(1 - cos\left(\Omega_{n+1,n} t_{BSB}\right) e^{-t_{BSB}/\tau}\right) \tag{S1}$$

where $P_{\uparrow}(t_{BSB})$ is the probability for the qubit state to be $|\uparrow\rangle$ at t_{BSB}, $p_{Y,n}$ is the phonon-n state population of the Y mode, $\Omega_{n+1,n}$ is the blue sideband Rabi frequency, and τ is the coherence time. The phonon population distribution is inferred by fitting the blue sideband Rabi oscillation data to Eq. (S1). When fitting the data, we set the maximum phonon number in the model, N, to 8 for $R = -2/3$ and to 12 for $R = -2$ to prevent overfitting. For all data points, the initial guess for the phonon population distribution is set to that of an even cat state. Also, we set the maximum amplitude of the Rabi oscillation to 0.97, because the detection error for the $|\uparrow\rangle$ state using the threshold method is 3% in our system, although changing it to 1.0 changes the results in Fig. 3 of the main text negligibly.

Lastly, the initial guess for the carrier Rabi frequency, Ω_0, which is the initial guess for the least square algorithm that extracts phonon number distribution from blue sideband Rabi oscillation, had to be chosen carefully because assuming an incorrect Rabi frequency

* taehyun@snu.ac.kr

will give you an inaccurate distribution. We consider the carrier Rabi frequency because the fitting function converts it into blue sideband Rabi frequency by multiplying it with relevant factors including the Y axis Lamb-Dicke factor, η_Y. We chose the initial Rabi frequency that gives the highest phonon population for the $|0\rangle_Y$ state for the $t_{SDF} = 0$ μs data. Throughout a single data set, the fitted Rabi frequency varied by less than 5%, as shown in Fig. S1.

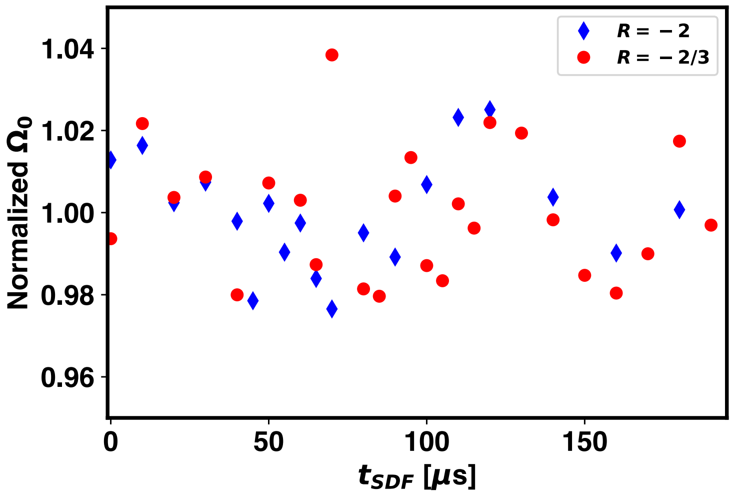

FIG. S1. Values of the fitted Ω_0 for the two data sets in Fig. 3 in the main text. The fitted Rabi frequencies vary less than 5 %, as expected.

The fitted values and standard errors for the parity modulation in Fig. 3 of the main text are as follows:

	$R = -2$	$R = -2/3$
$\Omega_{SDF}/(2\pi)$ [kHz]	167.683 ± 17.157	212.600 ± 6.389
$p_{X,1}$	0.287 ± 0.073	0.213 ± 0.074
$p_{Y,1}$	0.043 ± 0.024	0.056 ± 0.015

TABLE S1. Results of parity fitting obtained from Fig 3. (d) and (e) of the main text

II. SEQUENCES OF t_{SDF} USED FOR ENTANGLED COHERENT STATE EXPERIMENTS

The data collection process for each data point in Fig. 3(d) and (e) took about five minutes, thus the total data collection time for each of the two data sets spanned about two hours. To prevent the slow drift of the trap frequency affecting the observed variation in phonon number parity, we randomized the data taking sequence as presented below:

Sequence	1	2	3	4	5	6	7	8	9	10	11	12	13	14	15	16	17	18	19	20	21	22	23
$R = -2/3$<br>$t_{SDF}[\mu s]$	110	0	120	190	20	65	60	115	10	80	140	100	50	160	70	150	130	105	90	40	30	170	180
$R = -2$<br>$t_{SDF}[\mu s]$	65	110	90	70	100	55	60	40	30	80	0	120	10	50	45	20	160	180	140				

TABLE S2. Values and randomized sequences of t_{SDF} used to obtain experimental data in Fig. 3 (d) and (e) of the main text

III. SINGLE-MODE CAT STATE

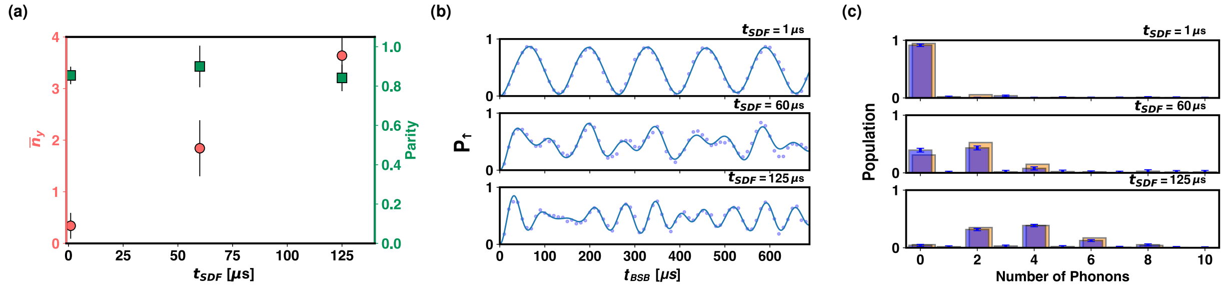

FIG. S2. **Single mode cat state analysis**. (a) Phonon number fitting results for a single-mode cat state in which only the Y motion is excited. The parity of the phonon number state remains high while the mean phonon number increases. (b) Blue sideband Rabi oscillation of the Y mode at various t_{SDF}. Solid lines are fits to phonon distribution model with a maximum phonon number of 10. (c) Phonon number distribution for each t_{SDF} extracted by fitting (b) to Eq. (S1). Blue bars are the experimentally measured phonon population and orange bars are the theoretically expected phonon population for a single-mode cat state for the measured mean phonon number. the extracted population matches that of a single-mode even cat state where odd number populations are suppressed. All error bars in this figure indicate standard errors of fitted parameters.

To ensure that our system and analysis scheme can accurately generate and correctly characterize non-classical states, we first created a single mode cat state ($|\psi_Y(t)\rangle = |\downarrow\rangle(|\beta(t)\rangle + |-\beta(t)\rangle)/\sqrt{2+2e^{-2|\beta(t)|^2}}$) by driving 1D SDF with the Y mode [3]. The results are presented in Fig. S2.

IV. POPULATION UNDER MØLMER–SØRENSEN INTERACTION

The populations of the qubit states in a two-ion chain evolve under Mølmer–Sørensen interaction with four motional states as follows:

$$P_{\downarrow\uparrow}(t) = P_{\uparrow\downarrow}(t) = \frac{1}{8}(2 - e^{-8\left((\overline{n}_1+\frac{1}{2})|\alpha_1(t)|^2+(\overline{n}_3+\frac{1}{2})|\alpha_3(t)|^2\right)} - e^{-8\left((\overline{n}_2+\frac{1}{2})|\alpha_2(t)|^2+(\overline{n}_4+\frac{1}{2})|\alpha_4(t)|^2\right)})$$

$$P_{\downarrow\downarrow}(t) = \frac{1}{8}(2 + e^{-8\left((\overline{n}_1+\frac{1}{2})|\alpha_1(t)|^2+(\overline{n}_3+\frac{1}{2})|\alpha_3(t)|^2\right)} + e^{-8\left((\overline{n}_2+\frac{1}{2})|\alpha_2(t)|^2+(\overline{n}_4+\frac{1}{2})|\alpha_4(t)|^2\right)}$$
$$+4\cos\left(4\Phi(t)\right) e^{-2((\overline{n}_1+\frac{1}{2})|\alpha_1(t)|^2+(\overline{n}_2+\frac{1}{2})|\alpha_2(t)|^2+(\overline{n}_3+\frac{1}{2})|\alpha_3(t)|^2+(\overline{n}_4+\frac{1}{2})|\alpha_4(t)|^2)})$$

$$P_{\uparrow\uparrow}(t) = \frac{1}{8}(2 + e^{-8\left((\overline{n}_1+\frac{1}{2})|\alpha_1(t)|^2+(\overline{n}_3+\frac{1}{2})|\alpha_3(t)|^2\right)} + e^{-8\left((\overline{n}_2+\frac{1}{2})|\alpha_2(t)|^2+(\overline{n}_4+\frac{1}{2})|\alpha_4(t)|^2\right)}$$
$$-4\cos\left(4\Phi(t)\right) e^{-2((\overline{n}_1+\frac{1}{2})|\alpha_1(t)|^2+(\overline{n}_2+\frac{1}{2})|\alpha_2(t)|^2+(\overline{n}_3+\frac{1}{2})|\alpha_3(t)|^2+(\overline{n}_4+\frac{1}{2})|\alpha_4(t)|^2)})$$

$$\Phi\left(t\right) = \sum_{n=1}^{4} \frac{\eta_{n1}\eta_{n2}}{(2d_n)^2}\left(d_n t - \sin\left(d_n t\right)\right)\Omega_0^2$$

where $n = 1, 2, 3$ and 4 is the index for the motional modes participating in the interaction corresponding to $X_{tilt}, X_{cm}, Y_{tilt}$ and Y_{cm} mode, respectively. $\alpha_n(t)$ is the phase space displacement of the n-th motional mode at time t, η_{nk} is the Lamb-Dicke factor for the n-th mode and the k-th ion, d_n is the laser detuning, $\overline{n}_n$ is the mean phonon number, and Ω_0 is the Rabi frequency. The above formulae were derived by following the calculations presented in [4]. These equations were used to analyze and derive the results about Mølmer–Sørensen interaction in the main text.

---

[1] Meekhof, D. M., Monroe, C., King, B. E., Itano, W. M. & Wineland, D. J. Generation of Nonclassical Motional States of a Trapped Atom. *Phys. Rev. Lett.* **76**, 1796–1799 (1996). URL https://link.aps.org/doi/10.1103/PhysRevLett.76.1796.

[2] Turchette, Q. A. *et al.* Decoherence and decay of motional quantum states of a trapped atom coupled to engineered reservoirs. *Phys. Rev. A* **62**, 053807 (2000). URL https://link.aps.org/doi/10.1103/PhysRevA.62.053807.

[3] Kienzler, D. *et al.* Observation of Quantum Interference between Separated Mechanical Oscillator Wave Packets. *Phys. Rev. Lett.* **116**, 140402 (2016). URL https://link.aps.org/doi/10.1103/PhysRevLett.116.140402.

[4] Manning, T. A. *Quantum information processing with trapped ion chains.* Ph.D. thesis, University of Maryland (2014).
